# The tibial bayonet method of wound closure

**DOI:** 10.1007/s11751-018-0304-3

**Published:** 2018-01-24

**Authors:** Peter O’Farrell, Annette-Christi Barnard, Franz Birkholtz

**Affiliations:** 1 Walk-A-Mile Centre for Advanced Orthopaedics, P.O. Box 11328, Centurion, Pretoria, 0046 South Africa; 2 Department of Orthopaedic Surgery, University of Pretoria, Pretoria, South Africa

**Keywords:** Limb salvage, Compound tibial fractures, Hexapod-assisted closure, Bayonet method, Duplication

## Abstract

Management of open lower limb fractures with soft tissue defects can be a technically challenging orthopaedic problem. Limited availability of orthoplastic services means that alternatives to the fix and flap concept are required in order to prevent infected non-unions from developing. The proposed ‘bayonet apposition’ allows the surgeon to temporarily shorten the limb without angulating the limb or creating a bone defect and removing viable bone. The viable bone edges are overlapped in a bayonet-like manner in order to appose the wound and skin edges. The limb length is restored by gradually distracting the bone segments once the soft tissues have healed. This is facilitated with a hexapod fixator for stabilization of the fracture and distraction. Prerequisites for utilizing this method are circumferential soft tissue damage to the lower limb with viable distal tissue. The bayonet method allows primary closure of a wound and rapid restoration of the native length of the limb.

## Introduction

Compound tibial fractures are severe limb-threatening injuries and can result in high patient mortality and protracted hospital care. These cases are often associated with significant soft tissue loss and defects that require both bony and soft tissue reconstruction with either regional or free tissue flaps [1]. The flaps are performed in conjunction with definitive fracture management.

Early soft tissue cover lowers infection rates by converting an open fracture to a closed one and has resulted in this ‘fix and flap’ concept being widely accepted as the preferred standard of care for compound tibias [2–6].

However, the limited availability of orthoplastic teams necessary to perform definite flaps or tissue transfers at the initial surgical setting results in a delay in soft tissue cover and increased post-operative infection rates [7]. The resultant higher morbidity in open fractures with delayed closure is well documented in the literature with infection rates up to 44% [8, 9].

The scarcity of combined orthoplastic teams has necessitated the development of alternative strategies for converting an open to a closed fracture. One of the alternatives is to create or exaggerate the deformity in order to close the wound primarily. There are a multitude of methods described that successfully utilize circular fixators (both Ilizarov fine-wire fixators and octahedral hexapod fixators) to close these defects primarily either by angulating and deforming the fracture or by acutely shortening the bone segment in order to close the wound [10–13]. Thereafter, the anatomical alignment of the bone is gradually restored by distraction osteogenesis or callotasis [12, 14–17]. This negates the need for moving soft tissue into the affected region. The downside of these procedures is a bone defect which either requires bone transport to correct the bone loss or a program to correct the deformity with associated complications such as joint stiffness and contractures [18].

**Fig. 1:**
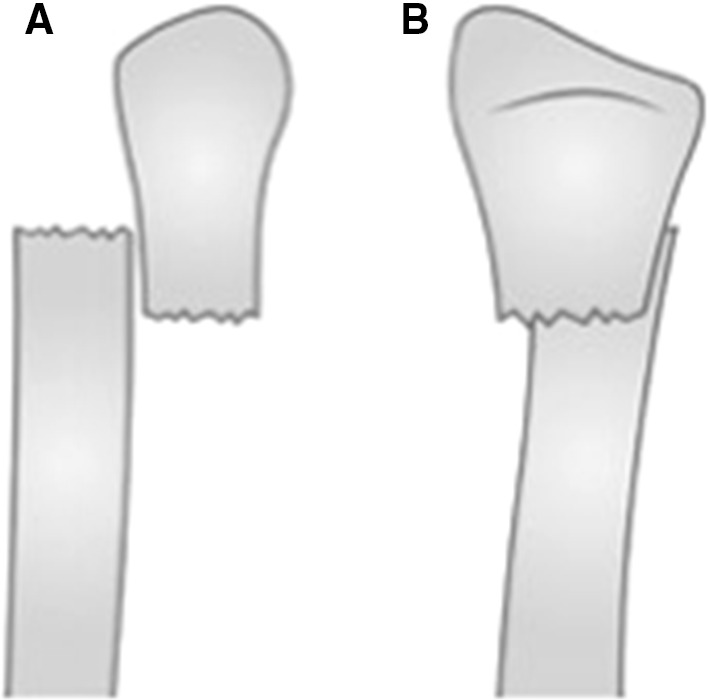
Line drawing of bayonet apposition to demonstrate the overriding of the bone segments, indicating the AP view (**a**) and lateral view (**b**)

This case report describes a novel, not previously described, method termed ‘bayonet apposition’ which allows the surgeon to temporarily shorten the limb without angulating the limb or creating a bone defect and removing viable bone. This is similar to the descriptive term as used to describe a distal radius deformity. The viable bone edges are overlapped in a bayonet-like manner in order to oppose the wound and skin edges [by definition, bayonet apposition is the relationship of two fracture fragments that lie next to each other rather than in end-to-end contact (Fig. 1)]. Once the soft tissues have healed, the length is restored by gradually distracting the bone segments and completing the program with a translational correction and compression at the fracture site.

There are several prerequisites for utilizing this method. Firstly, a transverse laceration extending more than two-thirds around the limb up to a completely circumferential laceration is required. Secondly, the soft tissues and bone need to have been adequately debrided, ideally performed by an experienced orthopaedic surgeon. This procedure can be done either at the initial setting in order to follow the concept of fix and flap surgery in converting an open fracture to a closed fracture or at the mandatory subsequent repeat visit to theatre following the initial debridement.

These are injuries that have a high complication rate osteomyelitis, bone loss, amputation, and the extent of the tissue damage needs to be appreciated by both the surgeon and the patient [19].

## Case report

### Patient

**Fig. 2:**
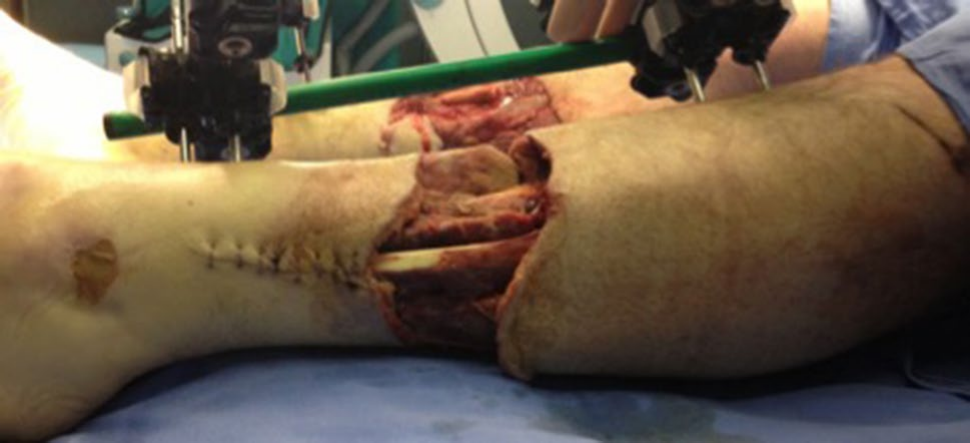
Extent of soft tissue injury following debridement awaiting definitive fixation

Patient was a 40-year-old male who sustained bilateral grade 3b tibia fractures (mechanism of injury: bark stripping machine at a sawmill) (Fig. 2). He had no other injuries and no co-morbidities.

### Technique and method

**Fig. 3:**
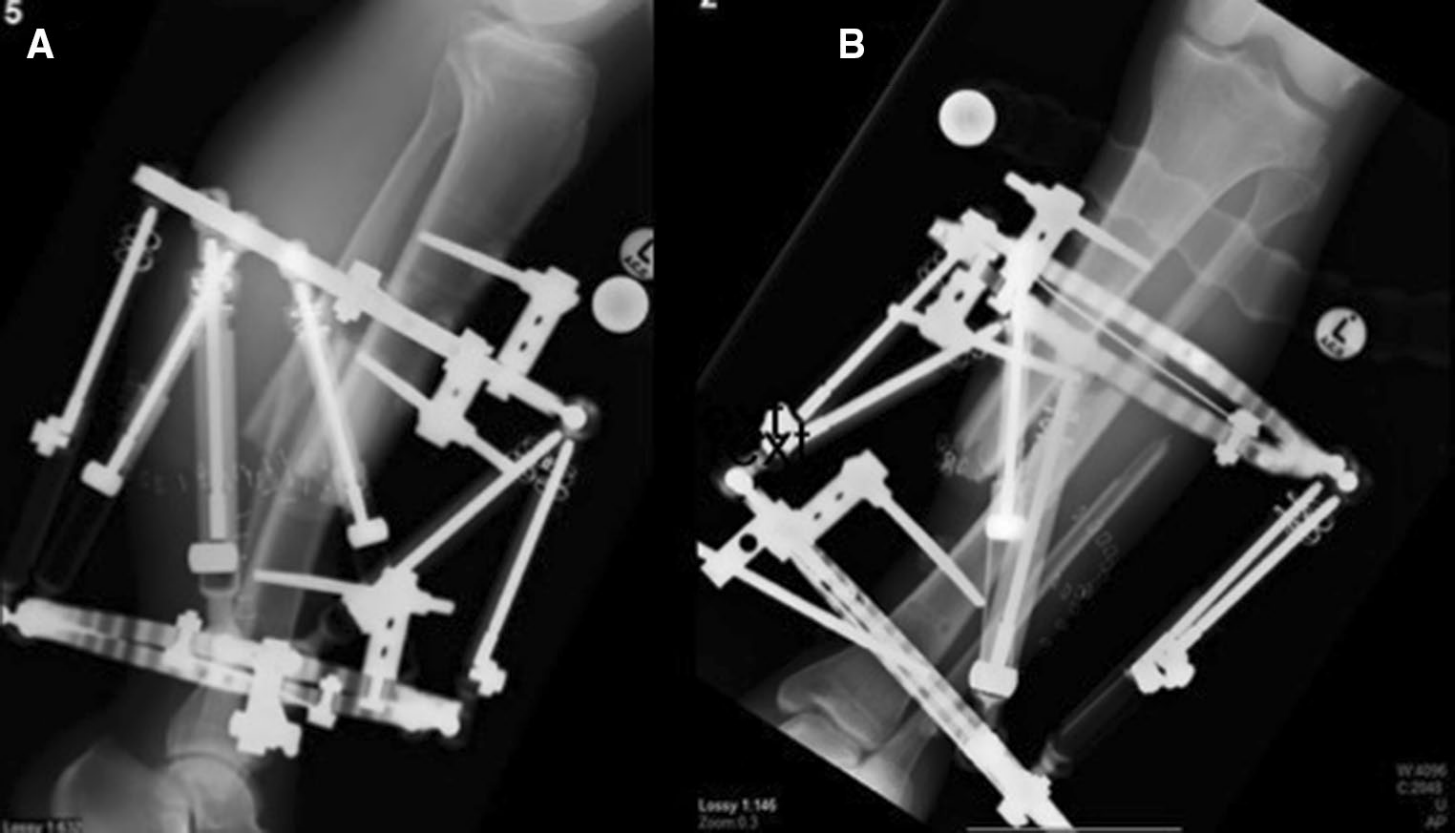
Two orthogonal views following the bayonet technique (post-operatively) lateral (**a**) and AP (**b**)

Following initial surgical management, a temporary fixator was applied along with appropriate debridement (Hoffmann Express Stryker^®^ comprising of four half pins and a standard two-bar configuration) (Fig. 3). The soft tissue defect was equivalent to a Gustilo–Anderson IIIB, and we were unable to have a flap applied [20].

Similar to the benchmark method of ‘fix and flap’ which usually includes two scheduled surgeries, the bayonet method too required two scheduled surgeries, however with the advantage of circumventing the need to be dependent on plastic surgeon’s availability: the first surgery for frame application, deformation and closure and the second for frame removal. In this case, there was also one unscheduled theatre visit in order to extend the frame across the ankle for the equinus correction.

**Fig. 4:**
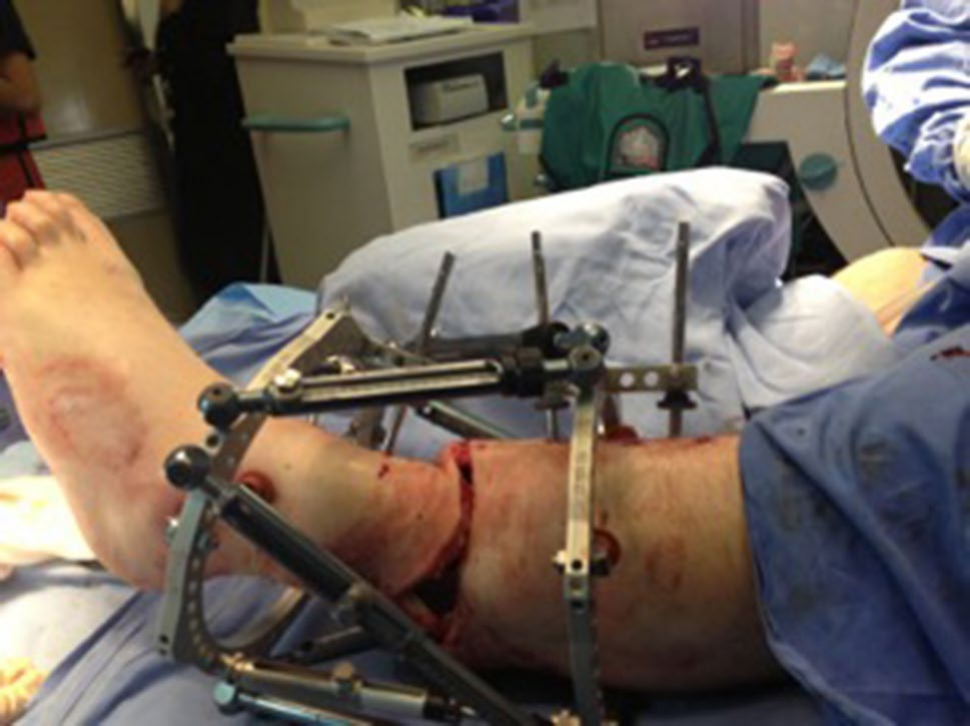
Soft tissue closure facilitated by bayonetting the tibia

At the 48 h, first definitive surgery a tibial bayonet procedure was performed, a technique that has been established at the Ilizarov Institute in Kurgan (AM. Cherkashin and ML. Samchukov, personal communication). Sub-muscular elevation and creation of a soft tissue envelope (no periosteal elevation or stripping) were performed (Fig. 4). At this sitting, a standard two-ring/four-half-pin hexapod frame was applied (OrthoFix TL-Hex^®^).

The wound was a 270-degree circumferential skin wound, from postero-lateral to postero-medial aspect of the middle third of the leg. The underlying muscle bellies were intact and did not require repair. Sensation and motor function were present distally (the latter was decreased). The struts were kept loose in order to allow bayonetting. The distal fragment was inserted into the soft tissue pocket. There was no interposing tissue, and the bone fragments were overlapped until the skin edges could be opposed in a tension-free manner (Fig. 4). The struts were locked in place, and the wound was closed primarily.

**Fig. 5:**
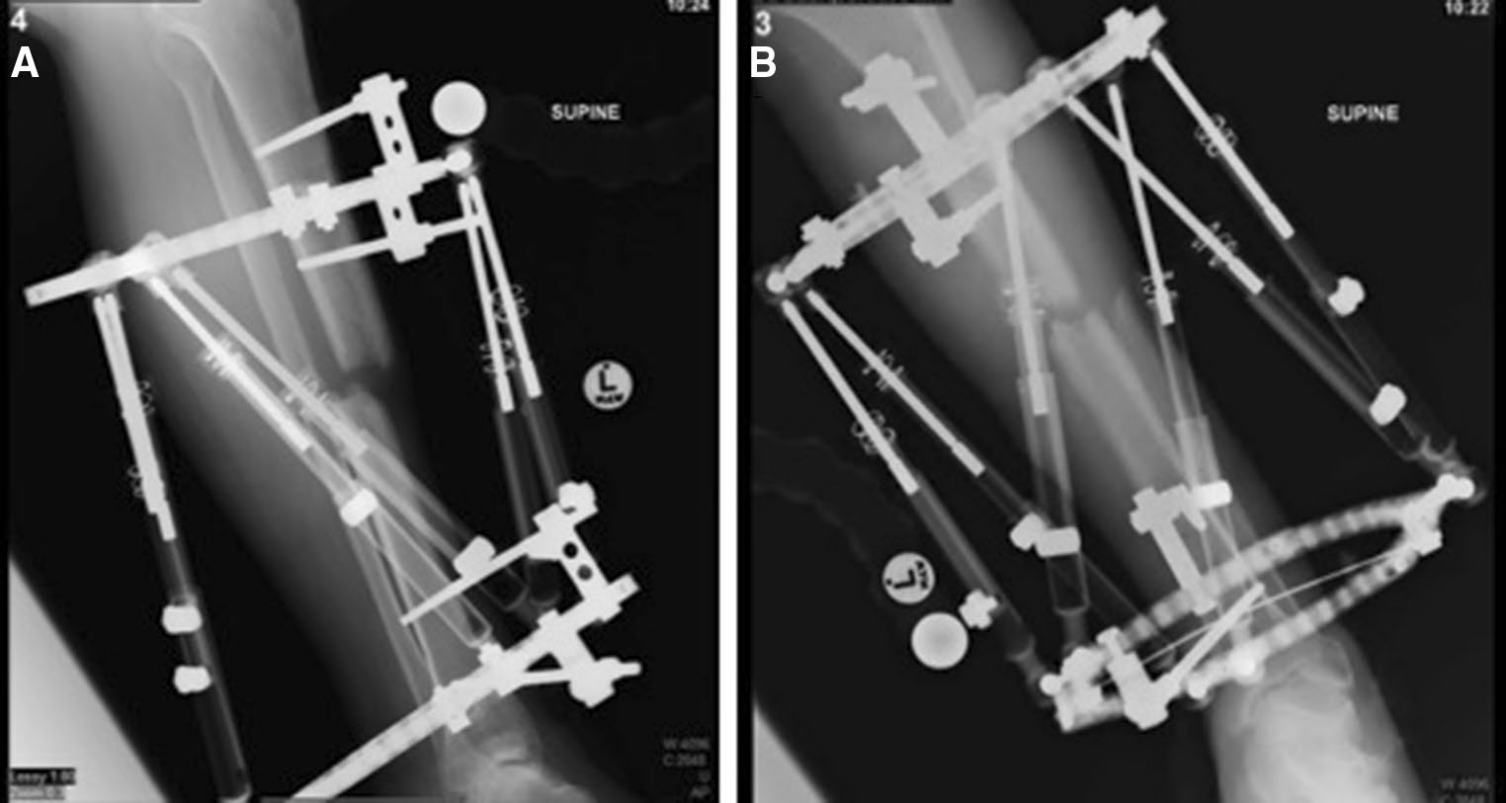
Bone segment alignment following distraction and realignment, lateral (**a**) and AP (**b**)

A latent period of 6 weeks followed with the foot elevated in order to allow the skin to heal and for the oedema to settle. Initial lengthening was achieved by increasing the length of all struts sequentially and equally. The distraction rate allowed a lengthening of 1–2 mm/day. Once length was restored, the TL-Hex^®^ software was used based on orthogonal radiographs for software referencing to allow realignment of the two fragments. Thus, the translation was corrected and the subsequent compression at the fracture site (Fig. 5). There was no formal docking procedure performed. The patient was assisted by the physiotherapists and mobilized with crutches and a walker frame within the first week falling surgery to allow full weight bearing with the aim to encourage functional mobilization. The accuracy of the correction was verified with serial radiographs at one monthly intervals.

## Results

**Fig. 6:**
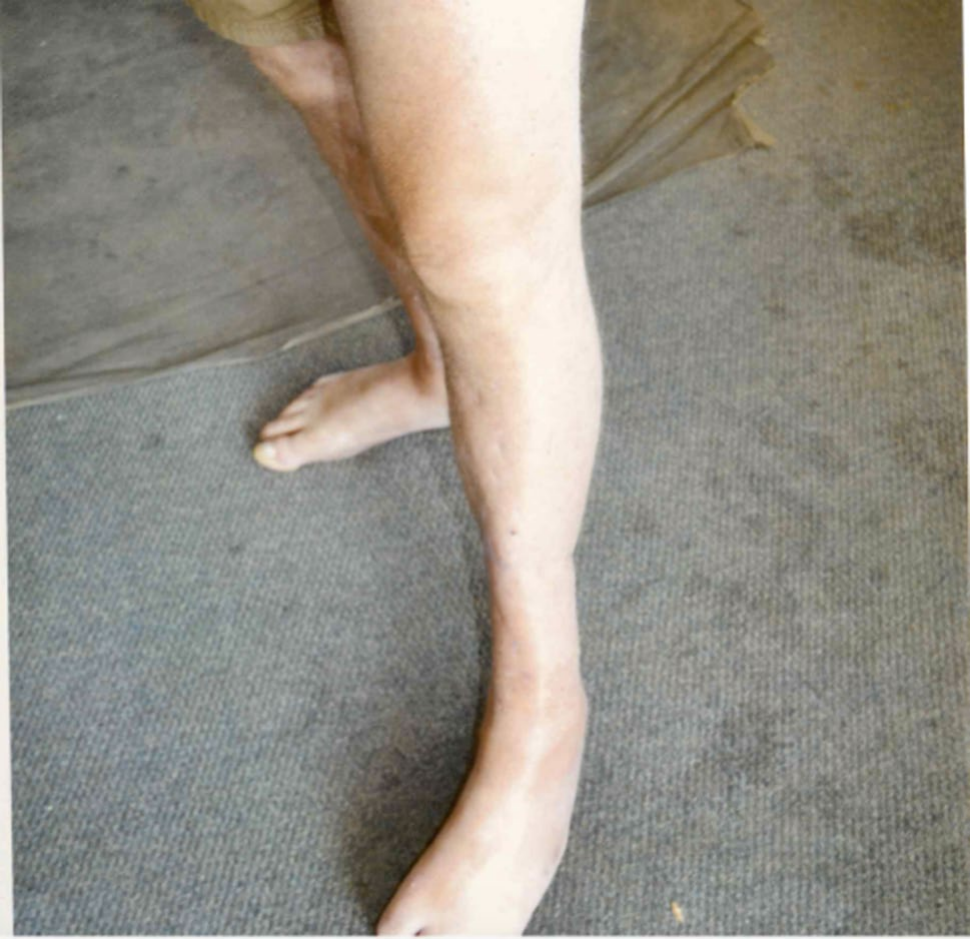
A final photograph of the healed limb, indicating that the soft tissue showed no signs of infection and the durability was good

The bayonet method described above allowed a potentially unsalvageable limb to be retained and managed in a technically easy manner. The standard trauma hexapod frame applied allowed the skin defect to be closed primarily. The correction took place over 2 months. The soft tissue and skin healed without any complications. With the final 2-year follow-up, the durability of the soft tissue was good with no signs of infection (Fig. 6).

**Fig. 7:**
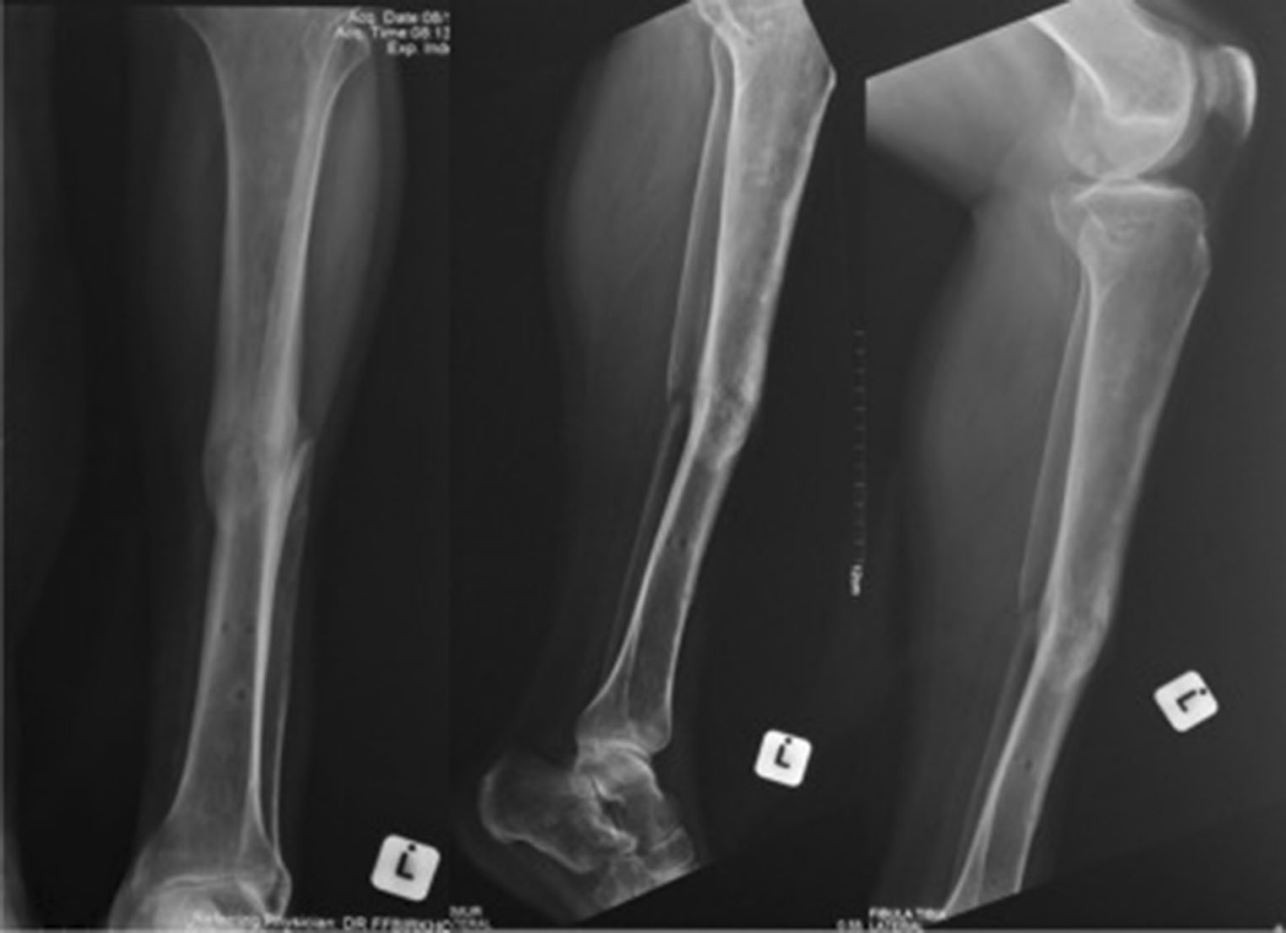
Radiograph series of 2-year follow-up. Fracture united and bone is aligned

Initially, there was eight centimetres of shortening. This was rapidly corrected in order to restore the length before contractions started developing. The patients total time in a frame was 11 months. This was made up of a 6-week latent period for skin healing followed by 2 months for correction. An equinus contracture developed after length and alignment was restored. This was corrected with a frame spanning the ankle. At the time of discharge, the patient had a leg that was equal in length to the contralateral side. Finally, at the time of the 2-year follow-up radiographs confirmed fracture union and that the bone was aligned (Fig. 7). The patient gained a satisfactory clinical and functional outcome.

## Discussion

Tibial shaft fractures with soft tissue defects are a technically challenging orthopaedic problem that require meticulous surgery and an array of skills necessary in order to prevent infected non-unions from developing. In optimal settings, free or local flaps are sufficient for definitive cover. There are acceptable associated risks and donor site morbidities associated with these free flaps. The limited availability of orthoplastic services prevents the flap and fix concept from being widely implemented.

Hexapod-assisted deformity creation allows soft tissue management with stable biomechanical fixation. This method has been successfully used in treating soft tissue defects in compound fractures by deforming and/or acutely shortening the affected limb [10, 12, 14]. The octatetrahedral hexapod utilizes a two-ring construct, with six struts and web-based program to correct deformities.

The bayonet method allows primary closure of a wound and rapid restoration of the native length of the limb. This avoids the need to be dependent on plastic surgeon availability for coverage of wounds. It is not the solution to all compound tibial fractures that need soft tissue cover but are another tool in the limb reconstruction quiver that enables the surgeon to facilitate patients to return to their premorbid functioning as quickly as possible.

Equinovarus foot is a known result of incorrect position while immobilizing a leg and problem with leaving leg short [21]. This could have been potentially avoided by a more aggressive restoration of the alignment and length. (Also, decreasing the ‘latent’ period could have prevented the subsequent deformity or lessened the severity.) If the adjustments had been started earlier, this morbidity could have been avoided. This could have also been avoided by spanning the ankle joint with the frame from the beginning.

Complications of an equinus contracture at the ankle and a caves foot are preventable and demonstrate the need to restore length early prior to musculo-tendinous units becoming contracted.

### Recommendations

While the formulation of a set of guidelines is beyond the scope of this paper, this is a viable option to treat circumferential wounds and tibial fractures that are not overtly contaminated and where minimal bone debridement is required.

Acute shortening should be avoided in cases where vascular compromise is a risk of if a vascular intervention or repair has occurred.

## Conclusions

Limb salvage is a rapidly developing/evolving field. Patients who were previously destined to have a limb amputated are now able to have that severely injured limb salvaged. While there are multiple methods to achieve this goal, every orthopaedic surgeon who engages in limb salvage work needs to add as many skills/tools to their armamentarium in order to take maximum advantage of available soft tissue and bone.
